# Effects of two-months balanced diet in metabolically healthy obesity: lipid correlations with gender and BMI-related differences

**DOI:** 10.1186/s12944-015-0131-1

**Published:** 2015-10-29

**Authors:** Mariangela Rondanelli, Chaterine Klersy, Simone Perna, Milena Anna Faliva, Gigliola Montorfano, Paola Roderi, Irma Colombo, Paola Antonia Corsetto, Marisa Fioravanti, Sebastiano Bruno Solerte, Angela Maria Rizzo

**Affiliations:** Department of Public Health, Experimental and Forensic Medicine, Section of Human Nutrition andDietetics, Azienda di Servizi alla Persona di Pavia, University of Pavia, Pavia, Italy; Service of Biometry & Clinical Epidemiology, Fondazione IRCCS “Policlinico San Matteo”, Pavia, Italy; Department of Pharmacological and Biomolecular Sciences, Laboratory of Membrane Biochemistry and Applied Nutrition, Università degli Studi di Milano, Milan, Italy; Department of Internal Medicine, Section of Gerontology and Geriatrics, Azienda di Servizi alla Persona di Pavia, University of Pavia, Pavia, Italy

**Keywords:** Balance diet, Desaturase activities, Fatty acid profile, Metabolically healthy obesity

## Abstract

**Background:**

Nowadays no researches has been performed on fatty acid profile (FA) and desaturase activity in metabolically healthy obesity (MHO). The aim of this study was to assessed gender and BMI-related difference in FA, estimated desaturase activities and the efficacy on metabolic changes produced by 2-months well-balance diet in MHO subjects.

**Methods:**

In 103 MHO subjects (30/73 M/F; age:42.2 ± 9.5) FA, estimated desaturase activity, body composition (by DXA), Body Mass Index (BMI), lipid profile, adipokines (leptin, adiponectin, grelin, glucagon-like peptide-1), insulin resistence (by Homestasis metabolic assessment), C-reactive proteine, Atherogenic index of plasma (AIP) and Body Shape Index (ABSI) have been assessed. Gender and BMI related difference have been evaluated and the efficacy produced by 2-months well-balance diet has been considered.

**Results:**

At baseline, obese subjects, compared to overweight, show a significantly higher oleic (*p* <0.050), monounsaturated fatty acids (*p* <0.040), C18:0 delta-9 desaturase activity (D9D) (*p* <0.040) and lower linoleic acid (*p* <0.020), polyunsaturated fatty acids (*p* <0.020) and n-6 LCPUFA (*p* <0.010). Concerning gender-related difference, women show a significantly higher arachidonic acid (*p* <0.001), polyunsaturated fatty acids (*p* <0.001), n-6 LCPUFA (*p* <0.002), and lower monounsaturated fatty acids (*p* <0.001), D6D activity (*p* <0.030), C18:0 D9D (0.000) and C16:0 D9D (*p* <0.030). The 2-months diet was associated with a significantly increase in arachidonic acid (*p* = 0.007), eicosapentaenoic acid (*p* = 0.030), docosahexaenoic acid (*p* <0.001), long chain omega 3 polyunsaturated fatty acids (n-3 LCPUFA) (*p* <0.001), delta-5 desaturase activity (D5D) (*p* = 0.002), glucagon like peptide-1 (*p* <0.001) and a significant decrease in palmitoleic acid (*p* = <0.030), n-6/n-3 LCPUFA (*p* <0.001), insulin resistance (*p* = 0.006), leptin (*p* = 0.006), adiponectin (*p* <0.001), grelin (*p* = 0.030), CRP (*p* = 0.004), BMI (*p* <0.001) and android fat mass (*p* <0.001).

**Conclusions:**

The balanced diet intervention was effective in improving metabolic indices.

## Introduction

Metabolically healthy obesity (MHO) is a phenotype related to a subgroup of obese individuals who do not have insulin resistance, lipid disorders or hypertension [1]. Selection criteria for MHO individuals were partially based on the National Cholesterol Education Program's Adult Treatment Panel III report (ATP III) for lipid profiles (triglycerides: ≤.7 mmol/l, total cholesterol: ≤5.2 mmol/l, HDL-cholesterol: ≥1.3 mmol/l and LDL-cholesterol: ≤2.6 mmol/l) and on the study of Karelis [2] for insulin sensitivity (HOMA < =1.95). When 4 out of 5 criteria are met, a diagnosis of the MHO individual could be made, as suggested by Karelis [2].

MHO is an important, emerging phenotype with disease risks somewhere intermediate between healthy, normal weight, and unhealthy, obese individuals. From a clinical perspective, if this subgroup of obese individuals really do not have increased risk of metabolic syndrome or cardiovascular disease (CVD), is still under discussion [1]. People from the MHO are apparently protected from metabolic complications of obesity and do not have higher risk of incident of chronic kidney disease [3].

On the contrary recent study have demonstrated in MHO an increased incident of fatty liver [4].

And Appleton and collaborators have demonstrated the development of cardiometabolic abnormalities in one-third of MHO studied subjects with a significantly increased risk of diabetes [5].

Conversely other authors indicates that MHO subjects have a CVD risks similar to those of normal-weight subjects [6, 7]. Some findings indicate that the CVD risk attributable to obesity requires the concomitant presence of metabolic risk factors and may be related to differences in body composition, fitness, and inflammatory profiles [8–10].

Studies in small selected samples of postmenopausal (cessation of menstruation for more than 1 year and a follicle-stimulating hormone level of at least 30 U/L) obese women also suggest that the MHO may have more favorable inflammatory profiles [11], less visceral fat, and possibly less hepatic fat [12] than their counterparts with insulin resistance and other metabolic abnormalities [13].

Finally Appleton demonstrated that MHO phenotype is transient and can move to metabolically at risk obese (MRO) one depending on life style and exposure to hazardous adipokines, indicating that target interventions might be useful to prevent MRO related risks.

On the contrary, there are no data on plasma fatty acid profile and desaturase activities in metabolically healthy obesity (MHO), and their possible role as MHO phenotype markers and associated risks of metabolic disorders.

In fact, various studies have demonstrated that insulin resistance and insulin resistant states are often associated with an alteration of fatty acid (FA) pattern in plasma, characterized by an increased proportion of palmitic (C16:0) and a low proportion of linoleic (C18:2 omega-6, LA) acids; the distribution of other fatty acids indicates also an increased activity of delta-9 and delta-6 desaturases [14].

Fatty acids play a key role in energy balance, carbohydrate and lipid metabolism, and regulation of gene transcription [15]. The plasma fatty acid profile is influenced by two factors equally important: dietary fat intake and endogenous fatty acid metabolism related also to desaturase enzymes, which regulate the degree of unsaturation of lipids throughout the body [16]. The delta-9 desaturase (D9D) catalyses the conversion of palmitic and stearic acids (C18:0) into monounsaturated fatty acid (MUFA), palmitoleic acid (C16:1) and oleic acid (C18:1, OA) respectively. It has been observed that delta-9 desaturase activity is high in conditions like diabetes, atherosclerosis, obesity and metabolic syndrome [17]. Delta-9 desaturase, also called stearoyl-CoA desaturase (SCD), is highly regulated and very sensitive to external changes, e.g., dietary factors and hormones. The changes in its activity are mirrored in the composition of cholesteryl esters, triglycerides and membrane phospholipids [18]. Delta-5 Desaturase (D5D) and delta-6 desaturase (D6D) are key enzymes in polyunsaturated fatty acids (PUFA) metabolism that catalyze the conversion of LA into arachidonic acid (C20:4 omega-6, AA) and that of alpha-linolenic acid (C18:3, omega-3, ALA) into eicosapentaenoic acid (C20:5 omega-3, EPA) and docosahexaenoic acid (C22:6 omega-3, DHA).

Gender differences have been reported in obese subjects for D9D, with higher activity in women than men. Moreover, women have been reported to have significantly higher levels of D6D activity than men [19].

Controlled fatty acid intake might influence plasma fatty acid profile and desaturase enzyme activities in obese subjects with typical metabolic disorders associated with obesity [20, 21]. Studies demonstrated that replacing saturated fatty acids in the diet with either MUFA [20] or PUFA [21] resulted in changes in serum fatty acid profile and improved insulin sensitivity.

Given this background, the aim of this study was to investigate if 2-months diet intervention in a group of metabolically healthy obese men and women is able to influence plasma fatty acid profile, estimated desaturase activities and adipokines. Moreover, the influence of gender and body mass index (BMI) on plasma FA pattern and desaturase activities was evaluated. For the study a 2 months dietetic intervention was chosen as it is known that the fatty acid composition in serum lipid esters mirrors to a certain extent the dietary fatty acids composition during the last 6–8 weeks [22, 23].

## Materials and methods

### Subjects

The study was performed following approval of the Ethic Committee of the Department of Internal Medicine and Medical Therapy, University of Pavia. Subjects gave their written consent to the study. All subjects had to give complete medical histories, and all underwent physical examination, anthropometric assessment and routine laboratory tests. Selection criteria for MHO individuals were partially based on the National Cholesterol Education Program's Adult Treatment Panel III report (ATP III) for lipid profiles (triglycerides: ≤.7 mmol/l, total cholesterol: ≤5.2 mmol/l, HDL-cholesterol: ≥1.3 mmol/l and LDL-cholesterol: ≤2.6 mmol/l) and on the study of Karelis [2] for insulin sensitivity (HOMA < =1.95). When 4 out of 5 criteria are met, a diagnosis of the MHO individual could be made, as suggested by Karelis [2].

Moreover, patients were excluded from the study if they met the Diagnostic and Statistical Manual-IV (DSM-IV) criteria for a current diagnosis of major depressive disorder as determined by the Structured Clinical Interview for DSM-IV Axis 1 Disorders (SCID-1) [24].

Subjects also were excluded if they were taking medications for weight loss, for control of cholesterol and TG, for anti-inflammatory purpose, or were pregnant or lactating, or if they had entered menopause. All participants agreed to refrain from participating in any other weight loss program. Alcohol intake, smoking habits and physical activity were recorded. Sedentary and non-smoking subjects, who did not drink more than 6 glasses (glass: 125 ml) of wine a week and did not drink hard liquor (alcohol content of at least 20 % Alcohol by volume), were admitted to the study.

### Procedures and study design

After 12 hours of fasting and abstinence from water since midnight, the subjects arrived at around 8:00 AM, using motorised transportation, at the Endocrinology and Clinical Nutrition Unit of Azienda di Servizi alla Persona di Pavia, University of Pavia (Italy).

Blood samples were taken for the routine measurements and in order to assess leptin, adiponectin, ghrelin, insulin, glycerol, total free fatty acid content. Body composition was determined by dual energy X-ray absorptiometry (DXA). The same evaluations were assessed after 2 months’ well balance intervention diet.

### Body composition measurement

Body composition was determined using a Lunar Prodigy DXA (GE Medical Systems, Waukesha, Wisconsin). The in vivo coefficients of variation were 4.2 and 0.48 % for fat and lean body mass respectively. Central fat (an approximation of visceral fat) was evaluated using always Dual-energy X-ray absorptiometry (DXA). This assessment consisted of measuring the fat percentage within a defined rectangle, from the upper edge of the second lumbar vertebra to the lower edge of the fourth lumbar vertebra. The vertical sides of this rectangular area were the continuation of the lateral sides of the rib cage [25].

#### Anthropometry, weight-loss program and food intake

Nutritional status was analyzed using anthropometric measurements at baseline and after 2 months in both groups. Body weight and height were measured and the Body Mass Index (BMI) was calculated (kg/m^2^). Skinfold thicknesses (biceps, triceps, suprailiac, subscapular) were measured twice using a Harpenden skinfold caliper at 5 min intervals at each site, following a standardized technique [26]. Sagittal abdominal diameter was assessed at the L_4–5_ level in the supine position and waist girth was also measured. Anthropometric variables were measured by a single investigator. Body weight reduction was induced by a low-energy mixed (55 % carbohydrates, 30 % lipids and 15 % proteins) diet providing 600 kcal less than individually estimated energy requirements based on the measured Resting Energy Expenditure (REE). The individual energy requirements were estimated by indirect calorimetry (ventilated hood system) at baseline and multiplied by a factor of 1.3, corresponding to a low physical activity level. The energy content and macronutrient composition of the diets adhered to the nutritional recommendations of the American Diabetes Association [27–29] with limit fat intake to <25 % of total calories (with limit saturated fat to <7 % of total calories and dietary cholesterol to <200 mg/day), protein intake to 15–20 % with 1.0 g kg body wt^−1^ day^−1^ and carbohydrate to 55–60 % with 14 g dietary fiber/1000 kcal.

These diets were designed to achieve weight losses of 0.5 to 1 kg per week and they are considered to be a low-risk intervention [28]. Individual diet plans were drawn up for each subject by the research dietitian. To optimize compliance, dietary instructions were reinforced each week by the same research dietician. Each consultation included a nutritional assessment and weighting. A 3-day weighed-food record of 2 weekdays and 1 weekend day was performed before the study and during the last week of intervention. One-day weighed-food records were completed in weeks 2, 5 and 7. Low energy diets and dietary records were analyzed using a food-nutrient database (Rational Diet, Milan, Italy). In order to assess compliance to the weight-reduction programme, a 24-hour dietary summary was assessed by the nutritionist at the end of the study.

### Biochemical analyses

Fasting venous blood samples were drawn between 8:00 and 10:00 AM in a sitting position. Blood collection and handling were carried out under strictly standardized conditions and in line with manufacturers’ recommendations. The blood samples required for Clinical Chemistry parameters were collected into evacuated tubes without anticoagulant, left for 1 h at room temperature, and then centrifuged for 15 min at 1500 × g. Following centrifugation, the serum was transferred into plastic tubes, rapidly frozen and stored at −80 °C until analysis (less than 1 month later). Moreover whole blood, collected into tubes with EDTA as anticoagulant, was used for haematological parameters. The C-reactive protein (CRP) was determined by Nefelometric High Sensitivity CRP (Dade Behring, Marburg, Germany).

Apolipoprotein (apo)A-I and apoB were enzymatically measured using an autoanalyzer (Hitachi, Tokyo, Japan).

To determine insulin resistance, subjects were instructed to fast for 12 h before blood was drawn. Furthermore, the subjects refrained from any kind of exercise for 48 h prior to the study. Female subjects were tested during the early follicular phase of their menstrual cycle (days 3–10). Insulin resistance was evaluated using the Homeostasis Model Assessment (HOMA) [30] using the following formula:

$$ \mathrm{HOMA}\hbox{-} \mathrm{I}\mathrm{R} = \left[\left(\mathrm{fasting}\ \mathrm{insulin},\ \upmu \mathrm{U}/\mathrm{mL}\right) \times \left(\mathrm{plasma}\ \mathrm{glucose},\ \mathrm{mmol}/\mathrm{L}\right)\right]/22.5 $$

Atherogenic index of plasma (AIP) [31], and Body Shape Index (ABSI) [32] have been calculated.

Serum concentrations of FFA and glycerol were determined by a quantitative colorimetric assay (BioAssay Systems, Hayward, CA); the minimum detectable concentrations are 0.007 mM/L and 0.01 mM/L respectively. Intra- and inter-assay coefficients of variation are <3 and <5 % respectively for both measurements.

Plasma acylated and unacylated ghrelin levels were measured using an enzyme immunometric assay (EIA) based on a double-antibody sandwich technique (BioVendor, Brno, CZECH REPUBLIC). Blood samples were drawn into a cold syringe and added to chilled Vacutainer® EDTA-plasma tubes, according to the manufacturer’s recommendations. The limit of detection for acylated ghrelin is 1.5 pg/mL (incubating for 20 h at +4 °C), and the intra- and inter-assay coefficients of variation are 7.5 and 8.3 % respectively. The minimum detectable concentration of unacylated ghrelin is 2 pg/mL (incubating for 20 h at +4 °C); the intra- and inter-assay coefficients of variation are 6.3 and 7 % respectively.

Serum adiponectin levels were measured using an enzyme-linked immunosorbent assay (ELISA) (R&D Systems, Inc., Minneapolis, MN, USA); the minimum detectable concentration is 0.246 ng/mL. The intra- and inter-assay coefficients of variation are 3.5 and 6.5 % respectively.

Serum leptin levels were measured using an enzyme-linked immunosorbent assay (ELISA) (R&D Systems, Inc., Minneapolis, MN, USA); the minimum detectable concentration is <7.8 pg/mL. Intra- and inter-assay coefficients of variation are 3.1 and 4.3 % respectively.

### Lipid analyses

Regarding plasma fatty acid profile, the analysis was carried out blind to the subject status. Plasma was stored at −80 °C until used for analysis. Lipids were extracted with different chloroform/methanol mixtures according to Folch [33] and fractionated by HPLC-ELSD and analyzed as previously describe [34, 35].

Fatty acid composition of whole plasma was determined by gas-chromatographic analysis. The fatty acid methylesters, obtained after trans-derivatization with sodium methoxide in methanol 3.33 % w/v, were injected into gas chromatograph (Agilent Technologies 6850 Series II) equipped with a flame ionization detector (FID) under the following experimental conditions: capillary column: AT Silar length 30 m, film thickness 0.25 μm, Gas carrier: helium, temperature: injector 250 °C, detector 275 °C, oven 50 °C for 2 min, rate of 10 °C min-1 until 200 °C for 20 min. A standard mixture containing methyl ester fatty acids was injected for calibration.

### Estimated desaturase activity

Desaturase activity was estimated indirectly as a product/precursor fatty acid ratio in serum phospholipids and cholesterol esters, which reflects both dietary intake and endogenous metabolism of fatty acids [16].

### Statistical analysis

Data on fatty acid profile and estimated desaturase activities were described as mean ± standard deviation (SD) or median and 25^th^-75^th^ percentiles (if skewed); they were compared between genders and between BMI groups (overweight/obese patients) with the unpaired Student *t* test or Mann Whitney U (if skewed) test. The mean difference between groups and its 95 % confidence interval (CI) were computed. Overall changes from baseline to the 2-months assessment were estimated with the paired Student *t* test or the sign test (if skewed); the mean (median) change was computed together with its 95 % CI. A multivariable regression model for repeated was fitted to control the change from baseline for gender and BMI group. Huber White robust standard errors were computed to account for intra-patient correlation.

Stata 13.1 (StataCorp, College Station, TX, USA) was used for computation. All tests were 2-sided. A p-value < 0.05 was considered statistically significant.

## Results

**Fig. 1:**
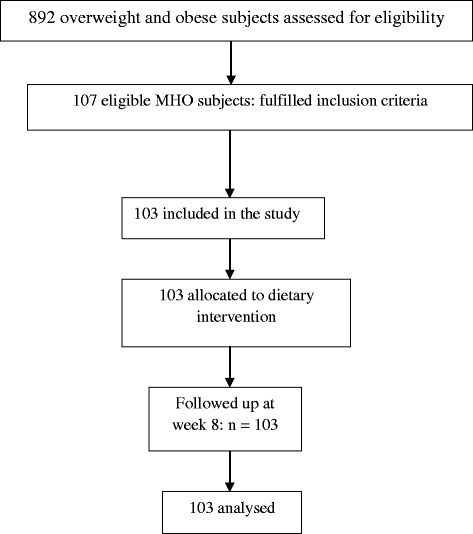
Flow diagram of the subjects studied

Eight hundred ninety two subjects were screened for eligibility. One hundred and three metabolically healthy obese subjects were included in the study, out of 107 eligible participants (Fig. 1). The baseline characteristics of the group are summarized in Table 1, together with their plasma fatty acid composition.

**Table 1:** Baseline characteristics of the studied group

Variable	MEAN ± DS
Age (years)	42.2 ± 9.5
BMI (Kg/m^2^)	30.2 ± 3.2
Fat mass (Kg)	33.9 ± 7.0
Free fat mass (kg)	45.8 ± 9.8
Android fat mass (%)	49.7 ± 6.1
apoA1 (g/L)	120.5 ± 25.8
apoB (g/L)	80.9 ± 24.9
Total-Cholesterol (mg/dl)	203.69 ± 37.53
HDL-Cholesterol (mg/dl)	52.64 ± 12.95
LDL-Cholesterol (mg/dl)	128.48 ± 31.8
Triglycerides (mg/dl)	112.87 ± 71.34
Glucose (mg/dl	89.96 ± 9.43
ABSI	6.12 ± 0.42
AIP	0.28 ± 0.27
Leptin (pg/mL)	218.6 ± 165.6
Adiponectin (ng/mL)	83.6 ± 49.6
Leptin/adiponectin	3.4 ± 3.5
GLP-1	1.3 ± 0.5
Grelin (pg/mL)	405.3 ± 253.3
C16:0	27.1 ± 2.6
C16:1	2.8 ± 0.9
C18:0	5.9 ± 0.8
C18:1	22.2 ± 3.6
C18:2 n-6	28.8 ± 4.4
C18:3 n-3	0.5 ± 0.2
C18:3 n-6	0.3 ± 0.2
C20:3 n-6	1.3 ± 0.3
C20:4 n-6	8.7 ± 1.7
C20:5 n-3	0.5 ± 0.3
C22:5 n-3	0.3 ± 0.2
C22:6 n-3	1.6 ± 0.5
AA/EPA	20.3 ± 10.2
Saturated fatty acids	33.1 ± 2.6
Monounsaturated fatty acids	24.9 ± 3.7
Polyunsaturated fatty acids (PUFA)	42 ± 5.02
Total omega-6 PUFA	39.1 ± 5.01
Total omega-3 PUFA	2.9 ± 0.8
omega-6/omega-3	14.3 ± 3.9

Plasma fatty acids are expressed as % of total content

*API* atherogenic index of plasma, *ABSI* body shape index, *CRP* c-reactive protein, *GLP-1* glucagon-like peptide-1, *HOMA* homeostatic model assessment

Table 2 shows the baseline gender differences in fatty acid profile and estimated desaturase activities. We observed that women, compared to men, show a significantly higher content of arachidonic acid (*p* = 0.0003), total polyunsaturated fatty acids (PUFA) (*p* = 0.0015), omega-6 PUFA (*p* = 0.0021), and a lower value of monounsaturated fatty acids (*p* = 0.0001), of delta-6 desaturase (*p* = 0.03), C18:0 delta-9 desaturase (*p* = 0.001) and C16:0 delta-9 desaturase (*p* = 0.03) activities.

**Table 2:** Baseline gender differences in fatty acid profile (% of total) and estimated desaturase activities

Variable	F	M	Δ (95 %)	*P* value
C16:0	26.9 ± 2.5	27.6 ± 2.8	−0.65 (−1.76, 0.46)	0.25
C16:1	2.8 ± 0.9	2.5 ± 0.7	0.30 (−0.08, 0.68)	0.13
C18:0	6.0 ± 0.8	5.7 ± 0.6	−0.29 (−0.04, 0.62)	0.09
C18:1	21.2 ± 2.8	24.6 ± 4.0	−3.33 (−4.70, −1.96)	**0.00**
C18:2 n-6	29.3 ± 4.3	27.5 ± 4.2	1.86 (0.01, 3.72)	**0.05**
C18:3 n-3	0.5 ± 0.2	0.5 ± 0.2	0.06 (−0.02, 0.14)	0.14
C18:3 n-6	0.3 ± 0.1	0.3 ± 0.2	−0.05 (−0.12, 0.01)	0.10
C20:3 n-6	1.4 ± 0.3	1.2 ± 0.3	0.16 (0.04, 0.28)	0.09
C20:4 n-6	9.1 ± 1.5	7.8 ± 1.9	1.32 (0.62, 2.02)	**0.0003**
C20:5 n-3	0.5 ± 0.3	0.6 ± 0.4	−0.05 (−0.17, 0.08)	0.45
C22:5 n-3	0.3 ± 0.2	0.3 ± 0.1	−0.001 (−0.07, 0.06)	0.97
C22:6 n-3	1.6 ± 0.5	1.5 ± 0.6	0.10 (−0.12, 0.32)	0.38
AA/EPA	21.2 ± 10.5	17.9 ± 9.0	3.32 (−1.02, 7.66)	0.13
Saturated fatty acids	33.0 ± 2.5	33.3 ± 2.8	−0.36 (−1.48, 0.75)	0.52
Monounsaturated fatty acids	24.1 ± 3.2	27.1 ± 4.1	−3.03 (−4.53, −1.53)	**0.0001**
Polyunsaturated fatty acids (PUFA)	43.0 ± 4.6	39.6 ± 5.2	3.40 (1.33, 5.46)	**0.0015**
Total omega-6 PUFA	40.0 ± 4.6	36.8 ± 5.3	3.29 (1.22, 5.35)	**0.0021**
Total omega-3 PUFA	3.0 ± 0.8	2.8 ± 1.0	0.11 (−0.24, 0.46)	0.55
omega-6/omega-3	14.4 ± 3.8	14.1 ± 4.2	0.25 (−1.44, 1.93)	0.77
Δ9 C16:0 Desaturase	0.1 ± 0.0	0.1 ± 0.0	0.01 (0.001, 0.03)	**0.03**
Δ9 C18:0 Desaturase	3.6 ± 0.7	4.4 ± 1.0	−0.75 (−1.10, −0.41)	**0.00**
Δ5 Desaturase	6.9 ± 1.9	6.6 ± 1.8	0.34 (−0.48, 1.15)	0.42
Δ6 Desaturase	0.0 ± 0.0	0.0 ± 0.0	−0.003 (−0.005, −0.0003)	**0.03**

Values in bold, *p* <0.05

The baseline differences in fatty acid profile and estimated desaturase activities between overweight (BMI < 30: 55 subjects) and obese (BMI > 30: 48 subjects) subjects are illustrated in Table 3. We observed that obese subjects, compared to overweight subjects, show a significantly higher value of oleic acid (C18:1) (*p* = 0.05), monounsaturated fatty acids (*p* = 0.03), C18:0 delta-9 desaturase activity (*p* = 0.04) and a lower value of linoleic acid (C18:2, LA) (*p* = 0.02), total PUFA (*p* = 0.02) and omega-6 PUFA (*p* = 0.01).

**Table 3:** Baseline differences in fatty acid profile and estimated desaturase activities in overweight and obese subjects

Variable	BMI < 30	BMI >30	Δ (95 %)	*P* value
C16:0	26.7 ± 2.3	27.6 ± 2.8	0.84 (−1.84, 0.16)	0.10
C16:1	2.6 ± 0.9	2.9 ± 0.9	−0.26 (−0.61, 0.08)	0.14
C18:0	6.0 ± 0.8	5.9 ± 0.7	0.16 (−0.15, 0.46)	0.31
C18:1	21.6 ± 3.3	22.9 ± 3.5	−1.36 (−2.72, 0.0018)	**0.05**
C18:2 n-6	29.7 ± 4.0	27.7 ± 4.5	2.06 (0.39, 3.73)	**0.02**
C18:3 n-3	0.5 ± 0.2	0.5 ± 2	0.03 (−0.04, 0.10)	0.42
C18:3 n-6	0.3 ± 0.2	0.3 ± 0.2	−0.0036 (−0.06, 0.06)	0.91
C20:3 n-6	1.4 ± 0.3	1.3 ± 0.3	0.04 (−0.07, 0.15)	0.43
C20:4 n-6	8.9 ± 1.5	8.5 ± 1.9	0.32 (−0.35, 1.00)	0.35
C20:5 n-3	0.5 ± 0.3	0.6 ± 0.3	−0.02 (−0.14, 0.1)	0.73
C22:5 n-3	0.3 ± 0.2	0.3 ± 0.1	0.03 (−0.03, 0.09)	0.27
C22:6 n-3	1.5 ± 0.4	1.7 ± 0.6	−0.16 (−0.36, 0.04)	0.12
AA/EPA	20.2 ± 9.5	20.3 ± 11.0	−0.09 (−4.08, 3.91)	0.97
Saturated fatty acids	32.7 ± 2.4	33.4 ± 0.8	−0.68 (−1.69, 0.33)	0.18
Monounsaturated fatty acids	24.2 ± 3.4	25.8 ± 4.0	−1.62 (−3.06, −0.19)	**0.03**
Polyunsaturated fatty acids (PUFA)	43.1 ± 4.5	40.8 ± 5.3	2.31 (0.38, 4.23)	**0.02**
Total omega-6 PUFA	40.2 ± 4.5	37.8 ± 5.3	2.42 (0.51, 4.34)	**0.01**
Total omega-3 PUFA	2.9 ± 0.7	3.0 ± 0.9	−0.12 (−0.44, 0.20)	0.47
omega-6/omega-3	14.8 ± 3.7	13.7 ± 4.0	1.06 (−0.46, 2.58)	0.17
Δ9C16:0 Desaturase	0.1 ± 0.03	0.1 ± 0.03	−0.01 (−0.02, 0.004)	0.21
Δ9C18:0 Desaturase	3.7 ± 0.8	4.0 ± 0.9	−0.34 (−0.68, −0.002)	**0.04**
Δ5 Desaturase	6.8 ± 1.9	6.8 ± 2.0	0.05 (−0.69, 0.80)	0.88
Δ6 Desaturase	0.01 ± 0.01	0.01 ± 0.01	−0.0007 (−0.002, 0.001)	0.47

Values in bold, *p* <0.05

Table 4 shows the macronutrient intake, assessed by 3-day weighed-food record of 2 week days and 1 weekend day, before and after of 2-months of well balanced intervention diet.

**Table 4:** Intake of macronutrients before and at the end of two-months of well balanced intervention diet

Daily nutritional intake	Pre treatment	Post treatment
Energy (kJ/day)	8985.3 ± 70.4	6792 ± 64.5
Protein (% of energy)	16.2 ± 1.0	17.5 ± 3.2
Carbohydrates (% of energy)	52.6 ± 2.1	54.4 ± 1.9
Fat (% of energy)	31.2 ± 2.1	26.1 ± 1.9
Saturated fatty acids (% of energy)	12.7 ± 2.9	7.1 ± 1.0
Cholesterol (mg)	213 ± 32.4	178.6 ± 30.5
Fiber (g/d)	16.8 ± 7.9	26.5 ± 3.6

Mean ± SD; nutritional evaluation carried out by Carnovale E. Marletta L. “Food composition tables”, Italian National Institute of Nutrition, Rome 1997

As shown in Table 5, after 2-months of intervention we observed a significant increase in all subjects of AA (*p* **=** 0.007), EPA (*p* **=** 0.03), DHA (*p* **<**0.001), omega-3 PUFA (*p* <0.001) content, and of delta-5 desaturase activity (*p* <0.05). Furthermore we demonstrated a significant decrease in palmitoleic acid (*p* **=** 0.038) and n-6 LCPUFA/n-3 LCPUFA ratio (omega-6/omega-3, *p* **=** 0.001).

**Table 5:** Fatty acid profile and estimated desaturase activities studied before and after 2-months of well-balanced diet intervention

Variable	Pre treatment	Post treatment	Δ (95 %)	*P* value	Multivariable *p* value^a^
C16:0	26.8 ± 2.4	26.6 ± 2.6	−0.16 (−0.69 to 0.35)	0.53	0.08
C16:1	2.7 ± 0.9	2.5 ± 0.9	−0.16 (−0.32 to −0.01)	**0.038**	**0.017**
			−0.20 (−0.37 to −0.04)^a^		
C18:0	5.9 ± 0.8	6.0 ± 0.9	−0.05 (−0.14 to 0.25)	0.59	0.93
C18:1	22.4 ± 3.6	22.3 ± 3.8	−0.10 (−0.86 to 0.65)	0.79	0.79
C18:2 n-6	28.7 ± 4.5	28.3 ± 3.9	−0.44 (−1.25 to 0.38)	0.28	0.22
C18:3 n-3	0.5 ± 0.2	0.5 ± 0.5	0.05 (−0.06 to 0.15)	0.38	0.43
C18:3 n-6	0.3 ± 0.1	0.3 ± 0.3	0.04 (−0.01 to 0.10)	0.14	0.09
C20:3 n-6	1.4 ± 0.3	1.3 ± 0.3	−0.04 (−0.09 to 0.00)	0.06	**0.019**
			−0.07 (−0.12 to −0.01)^a^		
C20:4 n-6	8.9 ± 1.6	9.3 ± 1.9	0.45 (0.12 to 0.77)	**0.007**	**0.001**
			0.64 (0.28 to 1.00)^a^		
C20:5 n-3	0.6 ± 0.3	0.7 ± 0.4	0.09 (0.01 to 0.17)	**0.030**	**0.007**
			0.11 (0.03 to 0.19)^a^		
C22:5 n-3	0.3 ± 0.2	0.3 ± 0.1	0.01 (−0.03 to 0.05)	0.69	0.33
C22:6 n-3	1.6 ± 0.5	1.9 ± 0.6	0.23 (0.13 to 0.33)	**<0.001**	**<0.001**
			0.29 (0.18–0.34)^a^		
AA/EPA	20.1 ± 10.4	19.4 ± 11.8	−0.62 (−2.80 to 1.55)	0.57	0.47
Saturated fatty acids	32.7 ± 2.4	32.6 ± 2.9	−0.11 (−0.74 to 0.51)	0.72	0.15
Monounsaturated fatty acids	25.1 ± 3.8	24.8 ± 3.9	−0.27 (−1.05 to 0.51)	0.50	0.79
Polyunsaturated fatty acids (PUFA)	42.2 ± 5.0	42.6 ± 4.7	0.38 (−0.65 to 1.41)	0.47	0.27
Total omega-6 PUFA	39.2 ± 5.1	39.2 ± 4.6	0.01 (−0.99 to 1.01)	0.99	0.81
Total omega-3 PUFA	3.0 ± 0.8	3.4 ± 1.0	0.37 (0.17 to 0.57)	**<0.001**	**<0.001**
			0.46 (0.26 to 0.66)^a^		
omega-6/omega-3	14.0 ± 4.0	12.6 ± 4.0	−1.37 (−2.16 to −0.57)	**0.001**	**<0.001**
			−1.67 (−2.47 to −0.88)^a^		
Δ 9C16:0 Desaturase	0.1 ± 0.0	0.1 ± 0.0	−0.01 (−0.01 to 0.00)	0.09	0.09
Δ9C18:0 Desaturase	3.9 ± 0.9	3.9 ± 1.1	−0.03 (−0.2 to 0.18)	0.77	0.74
Δ5 Desaturase	7.0 ± 1.9	7.8 ± 2.4	0.71 (0.35 to 1.08)	**<0.001**	**<0.001**
			0.96 (0.57 to 1.35)^a^		
Δ6 Desaturase	0.01 ± 0.01	0.01 ± 0.01	0.002 (−0.000 to 0.004)	0.10	0.06

^a^Adjusted for gender and BMI groups. Values in bold, *p* <0.05

The results shown in Table 6 confirm the efficacy of 2-months well-balance intervention diet on metabolic changes: we observed a significant increase of glucagon like peptide-1 (*p* **=** 0.000) and a significant decrease of insulin resistance, assessed by HOMA (*p* **=** 0.006), leptin (*p* **=** 0.006), adiponectin (*p* **=** 0.0001), grelin (*p* **=**0.03), C-reactive protein (*p* **=** 0.004), ABSI (*p* **=** 0.000), BMI (*p* **=** 0.000) and android fat mass by DXA (*p* **=** 0.000). These differences were maintained after adjusting for gender and BMI groups in the multivariable regression model. On the contrary, no significant correlations were found between the metabolic parameters measured.

**Table 6:** Hormones and body composition before and after 2-months of well-balanced diet intervention

Variable	Pre treatment	Post treatment	Δ (95 %)	*P* value
HOMA	1.97 (1.44 to 2.62)	1.74 (1.26 to 2.51)	−0.18 (−0.33 to 0.52)	**0.006**
CRP (mg/dL)	0.19 (0.11 to 0.35)	0.18 (0.09 to 0.31)	−0.04 (−0.06 to −0.01)	**0.004**
ApoA1 (g/L)	117.7 ± 25.9	119.9 ± 28.3	2.19 (−4.83 to 9.20)	0.54
ApoB1 (g/L)	79.4 ± 23.9	84.2 ± 21.7	4.87 (−0.36 to 10.10)	0.068
Total-Cholesterol (mg/dl)	202.5 ± 37.3	199.5 ± 38.9	−2.99 (−8.66 to 2.68)	0.30
HDL-Cholesterol (mg/dl)	52.28 ± 13.51	49.89 ± 12.75	−2.39 (−4.01 to 0.71)	**0.006**
LDL-Cholesterol (mg/dl)	127.76 ± 65.60	126.57 ± 68.00	−1.19 (6.30 to −3.92)	0.64
Triglycerides (mg/dl)	112.32 ± 74.08	115.26 ± 120.24	2.94 (−18.33 to 24.2)	0.78
Glucose (mg/dl)	90.45 ± 9.42	89.41 ± 9.96	−1.03 (−2.99 to 0.92)	0.29
ABSI	6.15 ± 0.43	6.04 ± 0.44	−0.10 (−0.14 to −0.06)	**<0.001**
AIP	0.28 ± 0.28	0.29 ± 0.29	0.01 (−0.03 to 0.04)	0.74
Leptin (pg/mL)	178.00 (97.00 to 311.20)	142.20 (80.50 to 250.10)	−17.30 (−31.45 to −5.35)	**0.006**
Adiponectin (ng/mL)	70.40 (50.60 to 101.70)	63.60 (42.30 to 96.30)	−7.35 (−11.20 to −3.80)	**0.0001**
Leptin/Adiponectin	1.94 (1.11 to 4.43)	2.04 (1.21 to 4.43)	85.00 (−0.26 to 0.25)	0.97
GLP-1 (pmol/L)	1.2 ± 0.5	2.0 ± 0.6	0.74 (0.54 to 0.95)	**<0.0001**
Ghrelin (pg/mL)	331.45 (216.40 to 516.50)	291.00 (188.30 to 449.60)	28.20 (−74.55 to 2.10)	**0.030**
Free fat mass (kg)	45.8 ± 9.8	45.7 ± 9.7	−0.10 (−0.44 to 0.23)	0.55
Android fat (%)	48.9 ± 6.1	47.3 ± 6.0	−1.58 (−2.12 to −1.05)	**<0.0001**
BMI (kg/m^2^)	29.8 ± 3.0	28.9 ± 2.9	0.89 (0.66 to 1.12)	**<0.0001**

*API* atherogenic index of plasma, *ABSI* body shape index, *CRP* c-reactive protein, *GLP-1* glucagon-like peptide-1, *HOMA* homeostatic model assessment. Values in bold, *p* <0.05

## Discussion

This study evaluated for the first time in the literature, the plasma fatty acid composition and the estimated desaturase activities, and their correlation with gender, obesity and diet intervention, in a population of metabolically healthy overweight and obese subjects. Until now, even the most recent studies on this topic have focused on subjects presenting or with increased metabolic syndrome [36] or diabetes probability [37].

The main result of this study is that 2-months of prudent diet intervention was associated with a significantly and independent improvement in metabolic indices, in particular a significant decrease in PCR, HOMA, n-6 LCPUFA/n-3 LCPUFA, leptin, grelin, palmitoleic acid and a significant increase in delta-5 desaturase activity, accompanied by a BMI and android fat mass reduction.

The improvement of these metabolic indices, secondary to the change in diet and weight loss, is in agreement to what was already demonstrated in the literature in obese subjects [38, 39]. Moreover, these changes were generally similar for men and women and for patients with higher and lower BMI, data not shown.

Our findings reinforce the hypothesis that also MHO subjects would benefit from a weight loss dietetic intervention.

With regard in particular to the plasma fatty acid profile, the palmitoleic acid decrease is an intriguing result of the study, since it is interesting to note that palmitoleic acid was also reported as a marker of cardiovascular disease risk [40].

Concerning fatty acid desaturase activity, the D6D and D9D indices were unaffected by diet intervention. The present observation regarding D6D agrees with previous reports by Vessby [41]. The increased serum D5D index found after diet intervention program is an attractive and positive result, since a high D5D index in serum predicts a reduced risk to develop the metabolic syndrome [42], and as well as cardiovascular death [43].

Previous study demonstrated that estimated desaturase activities are closely associated with the features of cardiometabolic risk in Koreans [43]. Our results on the other hand have not shown correlations between plasma fatty acid profile, estimated desaturase activities and metabolic indices, but this mismatch may be due to several factors: differences in the BMI of subjects studied (normal weight in the study by Do [44], overweight and obese subjects in the present study), and difference in ethnicity since significant ethnic differences has been found in desaturase activities [45].

The significant increase of arachidonic acid, eicosapentaenoic acid, docosahexaenoic acid, n-3 LCPUFA content, and a significant decrease of palmitoleic acid, n-6 LCPUFA/n-3 LCPUFA found in this study confirmed previous results indicating that serum lipids well reflect the dietary fat intake, other than is also influenced by desaturating enzymes [16].

Another innovative item of the study was a comparison of the plasma fatty acid profile and desaturase activities among the group of overweight and obese. We observed that obese subjects, compared to overweight subjects, show a significantly higher value of oleic acid, monounsatured fatty acids, activity of C18:0 delta 9 desaturase and a lower value of linoleic acid, polyunsaturated fatty acids and long chain omega 6 fatty acids.

Concerning fatty acid desaturases, the delta 9 desaturase activity (SCD-1) is increased in obese compared to overweight subjects, confirming data from literature: a high SCD-1 activity has been associated with obesity [46–48], and hypertriacylglycerolaemia [49].

Moreover, a high SCD-1 activity has been also associated with the metabolic syndrome [42], as well as with an increased risk to develop insulin resistance [41], cardiovascular diseases and death [43].

Concerning gender-related differences, we found that women, compared to men, show a significantly higher value of AA, total PUFA, omega-6 PUFA, and a lower value of MUFA, of delta-6 desaturase, C18:0 delta-9 and C16:0 delta-9 desaturase activities.

The higher arachidonic acid level in woman could be correlated to clinical observations and experimental findings in humans that already demonstrated that humoral and cell-mediated immune responses markedly differ between sexes [50]. For example, women mount stronger inflammatory and innate immune responses to bacterial and viral infections or to vaccination [51]. The more pronounced pro-inflammatory response pattern in women relative to men is generally thought to be beneficial by limiting pathogen spread and by accelerating pathogen clearance. However, inflammatory and autoimmune diseases are much more prevalent in women compared to men [52]. Our data on CRP indicate that the nutritional intervention is able to decrease inflammation in MHO subjects together with the omega-6 /omega-3 ratio that is considered a marker of inflammatory level related to the lipids mediators derived from these polyunsaturated fatty acids [53].

Gender differences have been previously reported in obese subjects also for delta-9 desaturase, with higher activity in women than men. Moreover, Warensjo et al. reported that women have significantly higher levels of delta-6 desaturase activities than men [19]. Our results are therefore in contrast to this previous study. However, the populations studied are very different (normal weight subjects involved in moderate physical activity for the research by Warensjo and sedentary MHO subjects in our study) and this could explain these differences.

The main limitation of our study is due to the lack of genetic factor assessment since studies on monozygotic twins have indicated that the conservation and distribution of fatty acids are subject to considerable genetic variance in humans [54].

Further studies are therefore needed on this specific MHO population, even going to compare the plasma fatty acid profile and desaturase activities of MHO subjects with non MHO group.

In conclusion, the prudent diet intervention was effective in improving metabolic indices and these findings reinforce the hypothesis that MHO subjects would also benefit from a lifestyle weight reduction program. Moreover, the high serum D5D index found after diet intervention program is an attractive result, since it predicts a reduced risk to develop the metabolic syndrome as well as cardiovascular death.

## Abbreviations

ATP III: National Cholesterol Education Program's Adult Treatment Panel III report
BMI: Body mass index
CRP: C-reactive protein
CVD: Cardiovascular disease
D5D: Delta-5 Desaturase
D6D: Delta-6 desaturase (D6D)
FA: Fatty acid
DXA: Dual-energy X-ray absorptiometry
HOMA: Homeostasis model assessment
MHO: Metabolically healthy obesity
MRO: Metabolically at risk obese
PUFA: Polyunsaturated fatty acids
REE: Resting energy expenditure
SCD: Stearoyl-CoA desaturase
